# Dual actions of Psalmotoxin at ASIC1a and ASIC2a heteromeric channels (ASIC1a/2a)

**DOI:** 10.1038/s41598-018-25386-9

**Published:** 2018-05-08

**Authors:** Yi Liu, Rebecca Hagan, Jeffrey Schoellerman

**Affiliations:** Neuroscience Discovery, Janssen Research & Development, L.L.C., 3210 Merryfield Row, San Diego, CA 92121 USA

## Abstract

Acid-Sensing Ion Channels (ASICs) are gated by extracellular protons and play important roles in physiological and pathological states, such as pain and stroke. ASIC1a and ASIC2a, two of the most highly expressed subunits in the brain, form functional homo- and hetero-meric (ASIC1a/2a) channels. The function of ASIC1a has been widely studied using psalmotoxin (PcTx1), a venom-derived peptide, as an ASIC1a-selective antagonist. Here, using whole-cell patch clamp, we show that PcTx1 has dual actions at ASIC1a/2a. It can either inhibit or potentiate the heteromeric channel, depending on the conditioning and stimulating pHs. Potent inhibition occurs only at conditioning pHs that begin to desensitize the channel (IC_50_ = 2.9 nM at pH7.0, a threshold pH for desensitization of ASIC1a/2a). By contrast, potent potentiation can occur at the physiological pH in both CHO cells (EC_50_ = 56.1 nM) and cortical neurons (threshold concentration < 10 nM). PcTx1 potentiates ASIC1a/2a by increasing the apparent affinity of channel activation for protons. As such, potentiation is the strongest at moderate pHs, diminishing with increasing proton concentrations. Our findings identify PcTx1 as a valuable tool for studying ASIC1a/2a function and contribute significantly to the understanding of the diverse and complex pharmacology of PcTx1.

## Introduction

Acid-sensing ion channels (ASICs) are cation channels activated by extracellular acidosis. At least four genes have been identified that encode six ASIC subunits: ASIC1a, ASIC1b, ASIC2a, ASIC2b, ASIC3, and ASIC4, among which, the “a” and “b” designations represent alternatively spliced variants of ASIC1 and ASIC2 genes, ACCN2 and ACCN1, respectively^1–3^. Each ASIC subunit has two transmembrane domains. Functional ASIC channels, which are sensitive to blockade by amiloride, are composed of three subunits assembled in either homomeric or heteromeric forms^4–6^.

ASIC1a and ASIC2a are highly expressed in the CNS with substantial overlap^7^. With an activation threshold near pH7.0, ASIC1a serves as a primary sensor of acidosis in the brain and is implicated in normal as well as patho-physiology, such as synaptic function/plasticity^8–11^, pain sensation^12–15^, psychiatric dysfunction^15^, seizure^16^ and neuronal injury^17–21^. ASIC2a appears only activatable at non-physiologically high proton concentrations, but has been shown to influence the localization of ASIC1a^22^. Much of the functional impact of ASIC2a may be through heteromerization with ASIC1a. Heteromeric ASIC1a/2a channels represent a significant fraction of ASICs in neurons^23–27^. As such and with an intermediate threshold pH for activation between ASIC1a and ASIC2a^4,26^, ASIC1a/2a may play important roles *in vivo*^7^.

Psalmotoxin (PcTx1), the prototypical ASIC1 modulator, is a 40 amino-acid peptide first isolated from the venom of the tarantula *Psalmopoeus cambridgei*. Although initially identified as a highly potent and selective inhibitor of homomeric ASIC1a^28^, PcTx1 has since been studied extensively on other ASIC channels and shown to also potently inhibit heteromeric ASIC1a/2b and ASIC1a/2a channels^26,29^. In addition, it has also been reported to potentiate and/or activate ASIC1a, ASIC1b and chicken ASIC1^30–33^. Thus, effects of PcTx1 on various ASIC1-containing channels can appear vastly different, depending on the gating properties of the channel and experimental conditions.

PcTx1 modulates both ASIC1a and ASIC1b by increasing the apparent affinity of channel gating for protons. Specifically, it shifts the pH dependence of steady-state desensitization (ASIC1a) and activation (ASIC1a and ASIC1b), respectively, towards lower proton concentrations^30,31^. Some evidence suggests that inhibition of ASIC1a/2b and ASIC1a/2a by PcTx1 shares this mechanism^26,29^. We hypothesized that this may be a more general mechanism by which PcTx1 modulates ASIC1-containing ASICs. If so, it may be possible to observe PcTx1 inhibition under certain conditions and potentiation under other conditions on the same channel for various ASIC1-containing ASICs.

In this study, we focused on ASIC1a/2a. We showed that PcTx1 conferred dual actions, inhibition and potentiation, on ASIC1a/2a, under conditions that paralleled those for its modulation of ASIC1a. Our findings further expand the diverse and complex pharmacology of PcTx1 at ASIC channels and contribute significantly to the understanding of the modulation of ASIC channels by PcTx1.

## Results

### Functional isolation of ASIC1a/2a stably expressed in CHO cells

**Figure 1:**
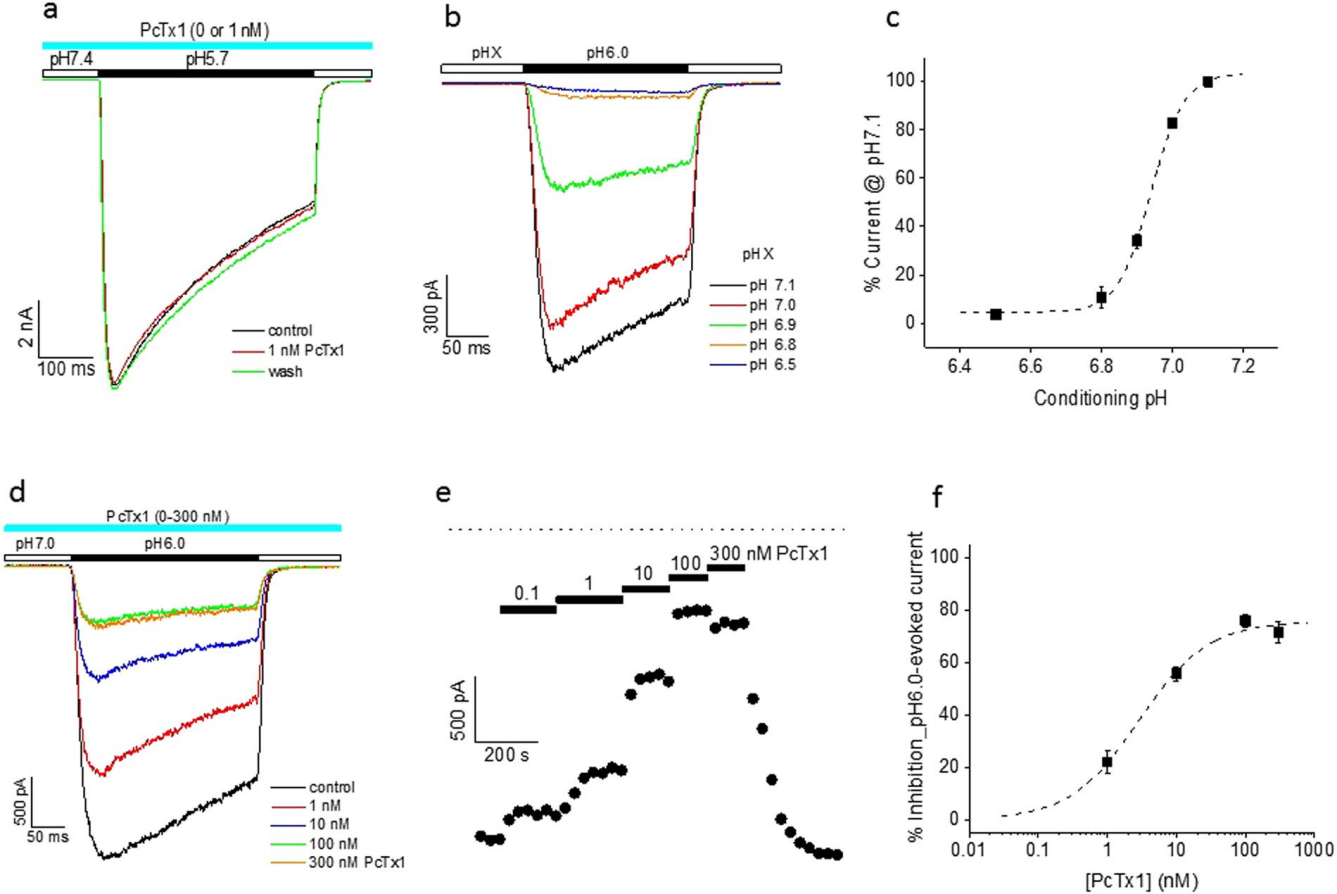
pH dependence of PcTx1 inhibition of ASIC1a/2a stably expressed in CHO cells. (**a**) Current traces from a single cell showing that PcTx1 (1 nM) had no effect on pH5.7-induced currents at the conditioning pH of 7.4. (**b**) Current traces from a single cell showing the dependence of pH6.0-induced current response on the conditioning pH as indicated. (**c**) Average (n = 5) of pH6.0-induced current amplitude (normalized to the value at conditioning pH7.1 for each cell before averaging) as a function of conditioning pH. pH_50_ = 6.94 from the best fit (dashed line). The holding potential was −80 mV. (**d**) Current traces from a single cell showing the inhibition of pH6.0-induced current at various PcTx1 concentrations (1–300 nM). The conditioning pH was 7.0. (**e**) Time course of PcTx1 modulation from a single cell. The dotted line at the top represents the current baseline. (**f**) Average % inhibition (n = 4–6) of pH6.0-induced current as a function of PcTx1 concentration. IC_50_ = 2.9 nM and the maximal inhibition was 75.7% from the best fit (dashed line).

**Figure 2:**
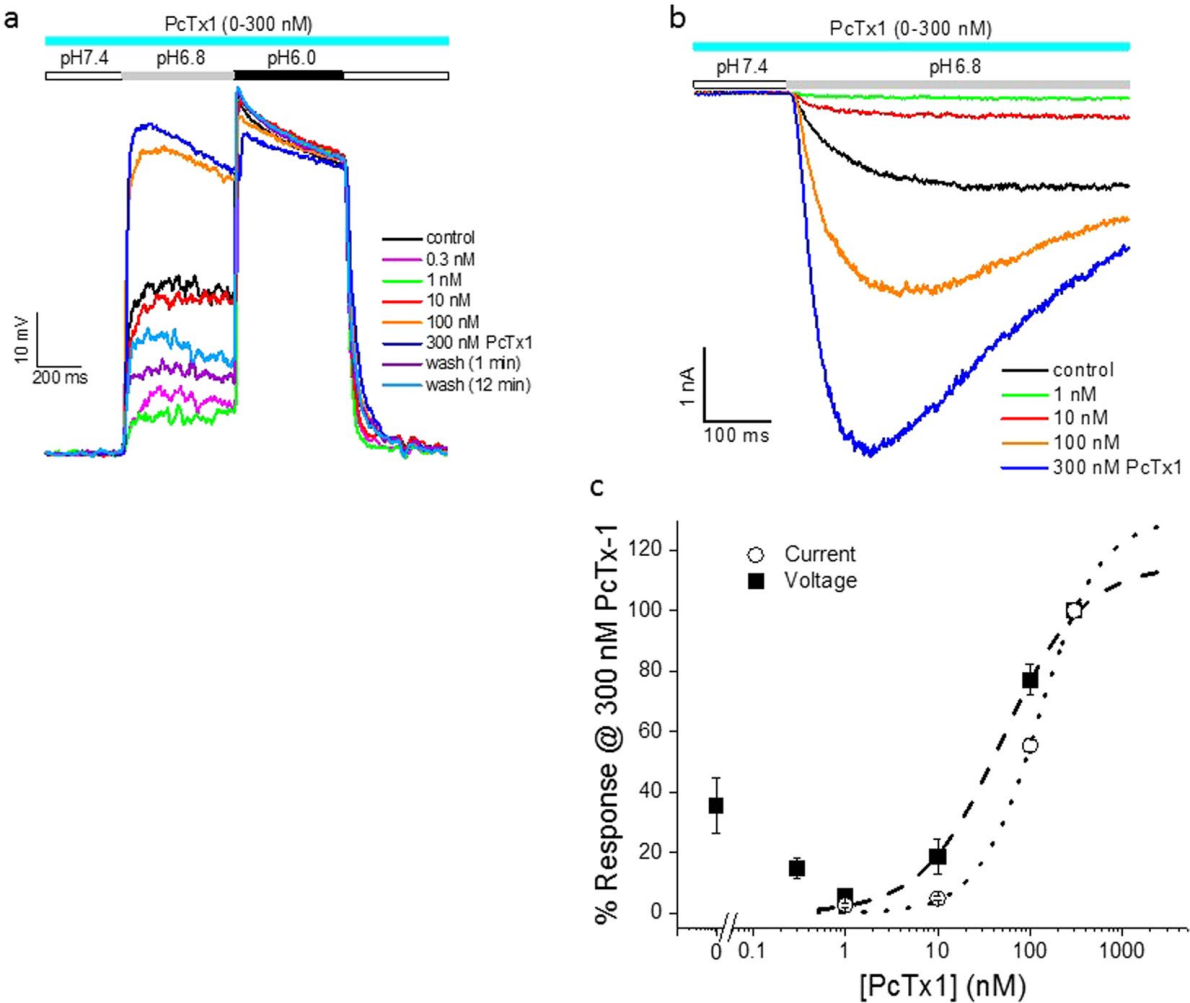
Potentiation by PcTx1 of ASIC1a/2a stably expressed in CHO cells. (**a**) Traces of pH6.8- and pH6.0-induced membrane depolarization from a single cell in the absence and presence of 0.3–300 nM PcTx1. PcTx1 was continuously applied in the order of increasing concentration till reaching steady state at each concentration. Note that the decrease in pH6.8-induced depolarization in the presence of 0.3 nM and 1 nM PcTx1 was reversed by 10–300 nM PcTx1. (**b**) Current traces from a single cell showing inhibition by 1 nM PcTx1 and potentiation by 10–300 nM PcTx1 of pH6.8-induced current responses. (**c**) Average % change in the amplitude of pH6.8-induced depolarization (solid squares; n = 7) and peak current (open circles; n = 6) as a function of PcTx1 concentration. Data were normalized to the value at 300 nM PcTx1 for each cell before averaging. Only data between 1 nM and 300 nM PcTx1 were used for curve fitting (dashed and dotted lines). IC_50_ values from the fits are 56.1 nM (depolarization) and 123.9 nM (current), respectively. The conditioning pH was 7.4 for all the experiments shown in Fig. 2.

**Figure 3:**
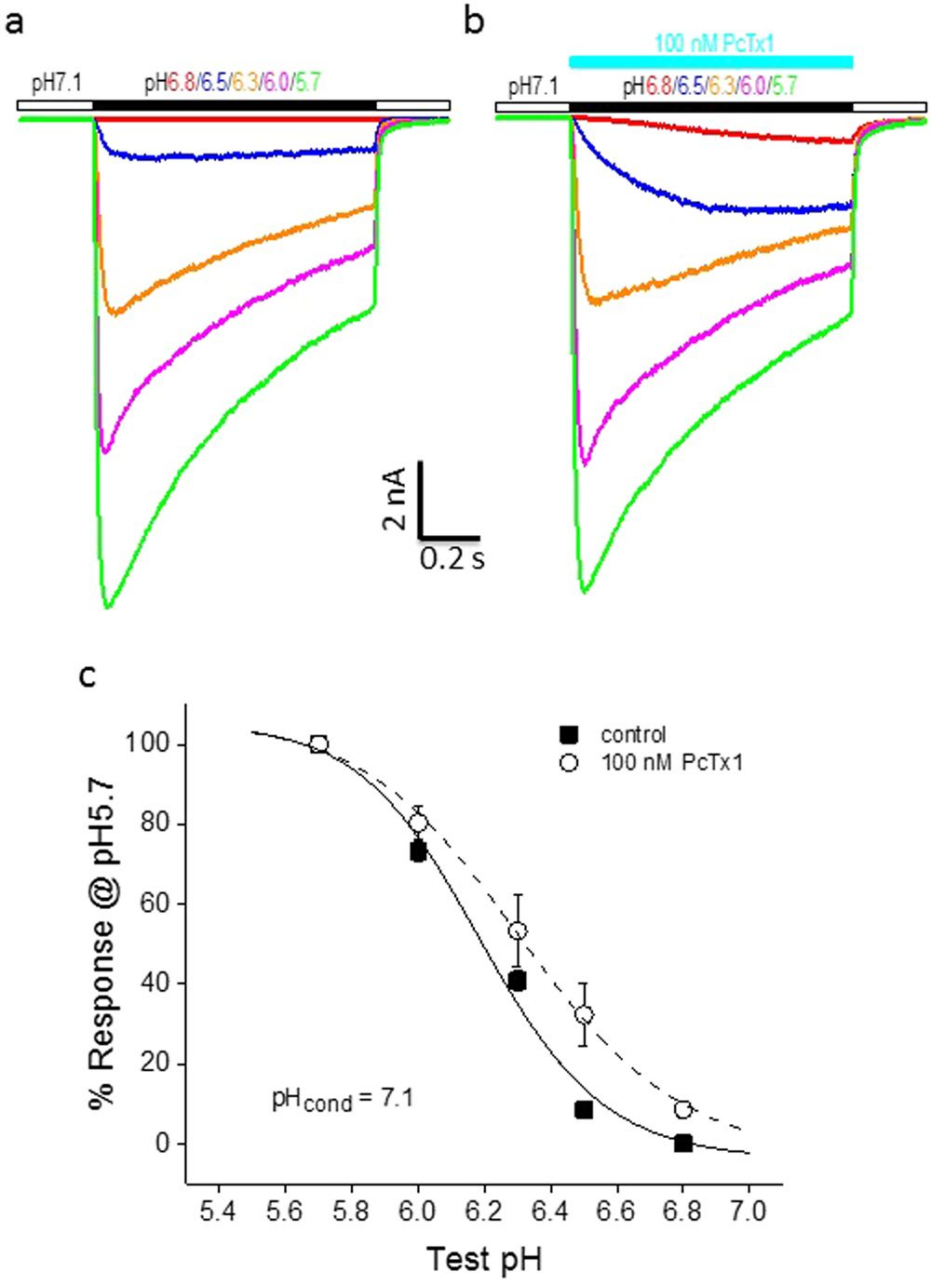
PcTx1 shifted pH activation of ASIC1a/2a toward lower proton concentrations in CHO cells. (**a**) Current traces from a single cell showing activation of ASIC1a/2a at various test pHs (pH6.8–5.7) in the absence of PcTx1. (**b**) Current traces from the same cell as in (**a**) showing activation of ASIC1a/2a at the same test pHs in the presence of 100 nM PcTx1. (**c**) Average current responses as a function of test pH in the absence (solid squares) and presence (open circles) of 100 nM PcTx1. Current amplitudes were normalized to that at pH5.7 for each cell in the absence (n = 6) and presence (n = 4) of PcTx1, respectively. The solid and dashed lines are best fits to the data in the absence (pH_50_ = 6.19) and presence (pH_50_ = 6.31) of 100 nM PcTx1, respectively. PcTx1 significantly (P < 0.05) shifted the pH activation curve to lower H^+^ concentrations. The conditioning pH was 7.1 for all the experiments in Fig. 3.

To study the effects of PcTx1 on ASIC1a/2a heteromeric channels, we first recorded from CHO cells recombinantly and stably expressing ASIC1a/2a. These cells also co-expressed ASIC1a and ASIC2a homomeric channels as well. We took the following steps to isolate only ASIC1a/2a heteromeric channels for investigation. Activation of ASIC2a was avoided by limiting the test pH to 5.7 or higher (see Supplementary Fig. S1). To remove functional contributions from ASIC1a, we took two different approaches. In one set of experiments, we held cells at pH7.0 or 7.1 (conditioning pH), taking advantage of the fact that, at these pHs, ASIC1a was fully desensitized (see Supplementary Fig. S2) whereas ASIC1a/2a was mostly not (Fig. 1c). In another set of experiments, where it was important to maintain the conditioning pH at 7.4, we exploited the selectivity of PcTx1 for ASIC1a over ASIC1a/2a (see Supplementary Figs S2 and 1a; also see ref.^26^) and the differential actions of PcTx1 at ASIC1a and ASIC1a/2a - inhibition of the former but potentiation of the latter at pH7.4 (Fig. 2). It should be noted that, except for experiments described in Figs 3 and S3, PcTx1 was included in both conditioning and test pH buffers.

### Inhibition of ASIC1a/2a by PcTx1 is pH dependent

We first studied the effects of low concentrations of PcTx1 on pH6.0- or pH5.7-induced currents in ASIC1a/2a-expressing CHO cells at the conditioning pH of 7.4. For these experiments, we selected cells that had relatively small pH6.8-induced currents arising from ASIC1a compared to pH6.0/pH5.7-induced currents, which, under these conditions, were primarily due to ASIC1a/2a activation. This ensured that the contribution of ASIC1a to the pH6.0/pH5.7-induced currents was negligibly small. As shown in Fig. 1a, PcTx1 had no effect on pH5.7-induced current at 1 nM. Data pooled from all cells recorded using pH6.0 and pH5.7 as the test pH showed that PcTx1 had no effect at either 1 nM (1.3 ± 0.9% inhibition, n = 5) or 10 nM (0.1 ± 2.6% inhibition, n = 5), in contrast to its significant inhibition of ASIC1a at these concentrations (see Supplementary Fig. S2). While pH7.4 was a desensitizing pH for ASIC1a (see Supplementary Fig. S2), significant desensitization for ASIC1a/2a only occurred at pH7.0 or lower (Fig. 1b and c). When we repeated the above experiments at the conditioning pH of 7.0, we found that the pH6.0-induced current was potently inhibited by PcTx1 in a concentration-dependent manner, with an IC_50_ value of 2.9 nM (Fig. 1d–f). As shown in Fig. 1e, the kinetics of this inhibition, both wash-on and wash-off, were quite rapid. Interestingly, PcTx1 inhibition of ASIC1a/2a was incomplete at high concentrations up to 300 nM, plateauing at ~75% (Fig. 1e and f).

### Potentiation of ASIC1a/2a by PcTx1 in CHO cells

Intrigued by the similarity in pH dependence between inhibition of ASIC1a and ASIC1a/2a by PcTx1, we were interested to see if PcTx1 might also mirror its other effect at ASIC1a and potentiate ASIC1a/2a as well. Potentiation of ASIC1a by PcTx1 was only evident at conditioning pHs that did not desensitize the channel^30^. We therefore conducted the next experiments at the conditioning pH of 7.4, a non-desensitizing pH for ASIC1a/2a. As would be expected, pH6.8 and pH6.0 buffers induced membrane depolarizations in CHO cells co-expressing ASIC1a and ASIC1a/2a recorded in the current-clamp mode (Fig. 2a). Most of the pH6.8-induced depolarization was inhibited by 1 nM PcTx1, consistent with the depolarization being elicited by activation of ASIC1a homomeric channels. In contrast, higher concentrations of PcTx1 (10–300 nM) caused larger, rather than smaller, pH6.8-induced depolarization than that in the presence of 1 nM PcTx1, with an EC_50_ value of 56.1 nM (Fig. 2c). This indicated that at these concentrations, PcTx1 potentiated ASIC1a/2a, since ASIC1a homomeric channels should be completely inhibited at these concentrations (see Supplementary Fig. S2). Similarly, pH6.8-induced currents were also potentiated by PcTx1 (Fig. 2b) at conditioning pH7.4 with an EC_50_ value of 123.9 nM (Fig. 2c). pH6.8 did not activate ASIC2a either in the absence or in the presence of 100 nM PcTx1, producing 0.5 ± 0.5% (n = 6) and 0.7 ± 0.6% (n = 5), respectively, of pH4.0-induced current in CHO cells stably expressing only ASIC2a homomeric channels. Therefore, PcTx1 potently and selectively potentiated ASIC1a/2a heteromeric channels under these conditions.

This potentiation resulted from PcTx1 shifting the pH dependence of ASIC1a/2a activation to lower H^+^ concentrations, promoting channel opening. As shown in Fig. 3, 100 nM PcTx1 significantly (P < 0.05) increased the apparent affinity of ASIC1a/2a for H^+^ from pH6.19 to pH6.31. As such, potentiation was most pronounced at moderate test pHs. It should be noted that in the experiments described before Fig. 3, PcTx1 was present in all the solutions, i.e., both in the conditioning pH solution and in the test pH solution(s). It was under these conditions that PcTx1 inhibited ASIC1a/2a at conditioning pH7.0. However, if PcTx1 was only co-applied with the test pH solution without preincubation, the resultant effect was potentiation even at pH7.0, at least at moderate test pHs (see Supplementary Fig. S3; although see Supplementary Fig. S4 for potentiation of residual current by preincubated PcTx1 even at conditioning pH7.0), probably because PcTx1 was present for too brief a period to significantly desensitize ASIC1a/2a. This co-application protocol was used in Fig. 3 so that potential complications from PcTx1 inhibition could be avoided when cells were held at pH7.1. Experiments using the preincubation protocol at conditioning pH7.4 also yielded results in general agreement with the conclusions arrived at in Fig. 3 (See Supplementary Table 1).

### Potentiation of acid-induced responses by PcTx1 in rat cortical neurons

**Figure 4:**
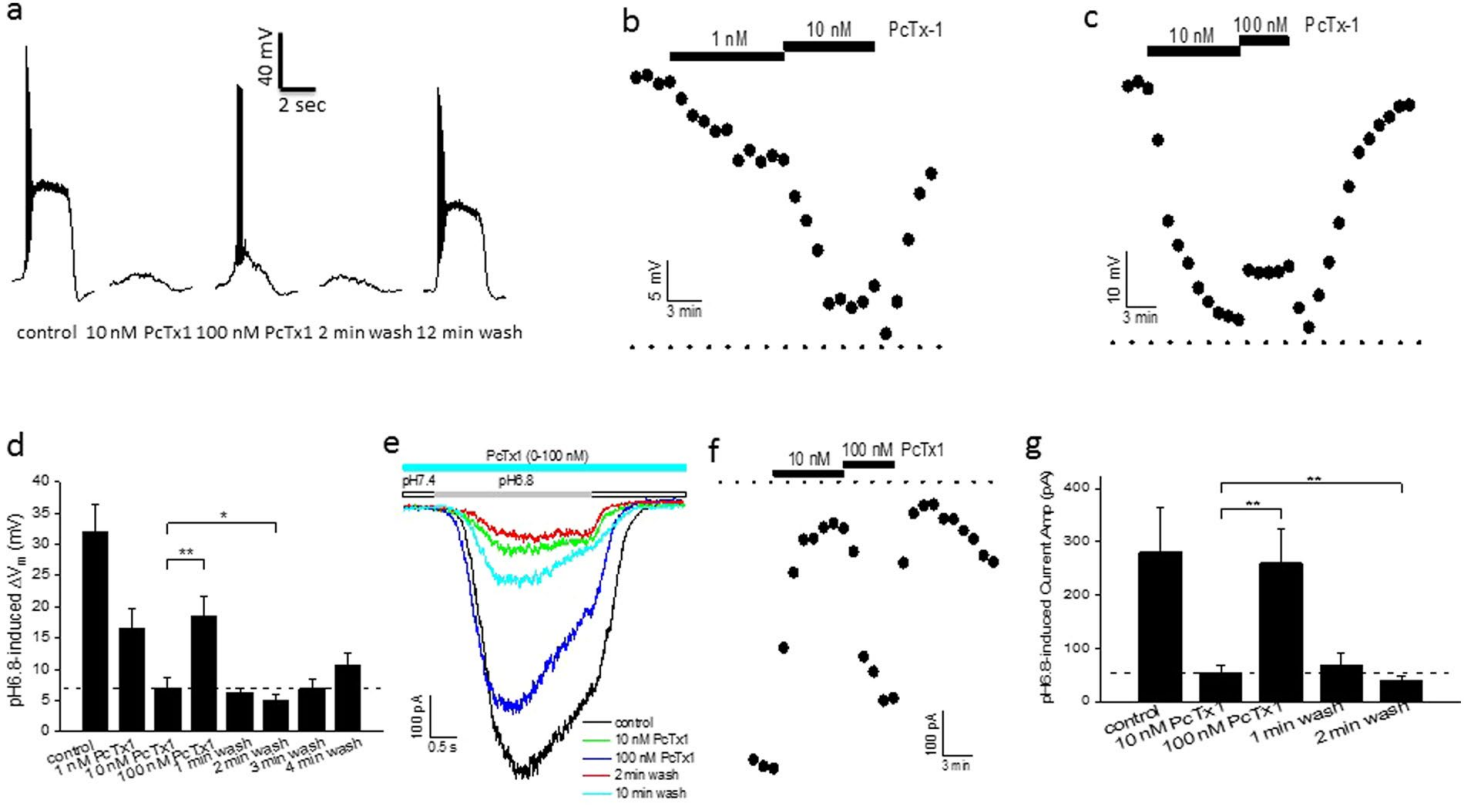
Potentiation of ASIC1a/2a by PcTx1 in rat cortical neurons. (**a**) Traces of pH6.8-induced membrane depolarization from a single neuron in the absence and presence of PcTx1. (b and c) Time courses of PcTx1 modulation for the same neuron as in (**a**). The dotted lines at the bottom represent the baseline. Note the dip in membrane potential to below that in the presence of 10 nM PcTx1 upon initial wash of 10 nM (**b**) and 100 nM (**c**) PcTx1. (**d**) Averages of pH6.8-induced depolarization from multiple neurons (n = 8, 4, 8 and 8 for 0, 1, 10 and 100 nM PcTx1, respectively). The horizontal dashed line represents the value in the presence of 10 nM PcTx1. PcTx1 was applied in the order of increasing concentration and the wash data were recorded upon wash-off of 100 nM PcTx1. (**e**) Traces of pH6.8-induced current from a single neuron in the absence and presence of PcTx1. (**f**) Time course of PcTx1 modulation from the same neuron as in (**e**). The dotted line at the top represents the baseline. Note the lower current amplitude than that in the presence of 10 nM PcTx1 upon first removal of 100 nM PcTx1. (**g**) Averages of pH6.8-induced currents from multiple neurons (n = 10 each for 0, 10 and 100 nM PcTx1, respectively). The horizontal dashed line represents the value in the presence of 10 nM PcTx1. PcTx1 was applied in the order of increasing concentration and the wash data were recorded upon wash-off of 100 nM PcTx1. For all the experiments in Fig. 4, the conditioning pH was 7.4 and the test pH of 6.8 was applied for 2–3 sec before returning to pH7.4. This pH application protocol was repeated once every 60 sec.

Next, we were interested to see if the potentiating effect of PcTx1 was just limited to an artificial expression system, or also occurred in neurons. Figure 4a provided the first evidence that the latter might indeed be the case. In this neuron recorded in the current-clamp mode, pH6.8 control buffer induced large depolarizations with action potentials superimposed in the rising phase of the depolarization. At 10 nM, PcTx1 inhibited both the action potentials and most of the depolarization. At 100 nM, however, PcTx1 partially restored the depolarization as well as action potentials. Figure 4b and c showed the time course of the PcTx1 actions on pH6.8-induced membrane depolarization in this neuron. At lower concentrations (1 and 10 nM), the predominant effect of PcTx1 was time- and concentration-dependent inhibition (Fig. 4b), presumably by inhibiting ASIC1a homomeric channels expressed in the neuron. At 100 nM PcTx1, however, the pH6.8-induced depolarization was rapidly, albeit partially, reversed (Fig. 4c). It is intriguing to note that the initial 1–2 min wash caused the depolarization to dip below that in the presence of 10 nM PcTx1 in both Fig. 4b and c. This suggested that (1) 10 nM was above the threshold concentration of PcTx1 for the potentiation, and (2) this potentiation was not only reversible, but also rapid relative to the recovery from ASIC1a inhibition. Prolonged wash restored the depolarization mostly to the pre-PcTx1 level, as the neuron recovered both from potentiation of ASIC1a/2a (faster) and from inhibition of ASIC1a (slower) by PcTx1. The data for the effects of PcTx1 on pH6.8-induced depolarization was summarized in Fig. 4d for all the neurons tested. In addition to membrane depolarization, we also examined the PcTx1 effects on pH6.8-induced currents in rat cortical neurons. As shown in Fig. 4e–g, effects of PcTx1 on pH6.8-induced currents in these neurons generally mirrored those on pH6.8-induced membrane depolarization. Note again the rapid decrease in current amplitude upon initial wash of 100 nM PcTx1 to below that at 10 nM PcTx1 (Fig. 4f) before the slow recovery from PcTx1 inhibition of ASIC1a. Preliminary data (see Supplementary Fig. S5) showed that this potentiation was further augmented by zinc and inhibited by amiloride, suggesting that ASIC1a/2a was likely the target.

### Potentiation of acid-induced responses by PcTx1 in mouse cortical neurons

**Figure 5:**
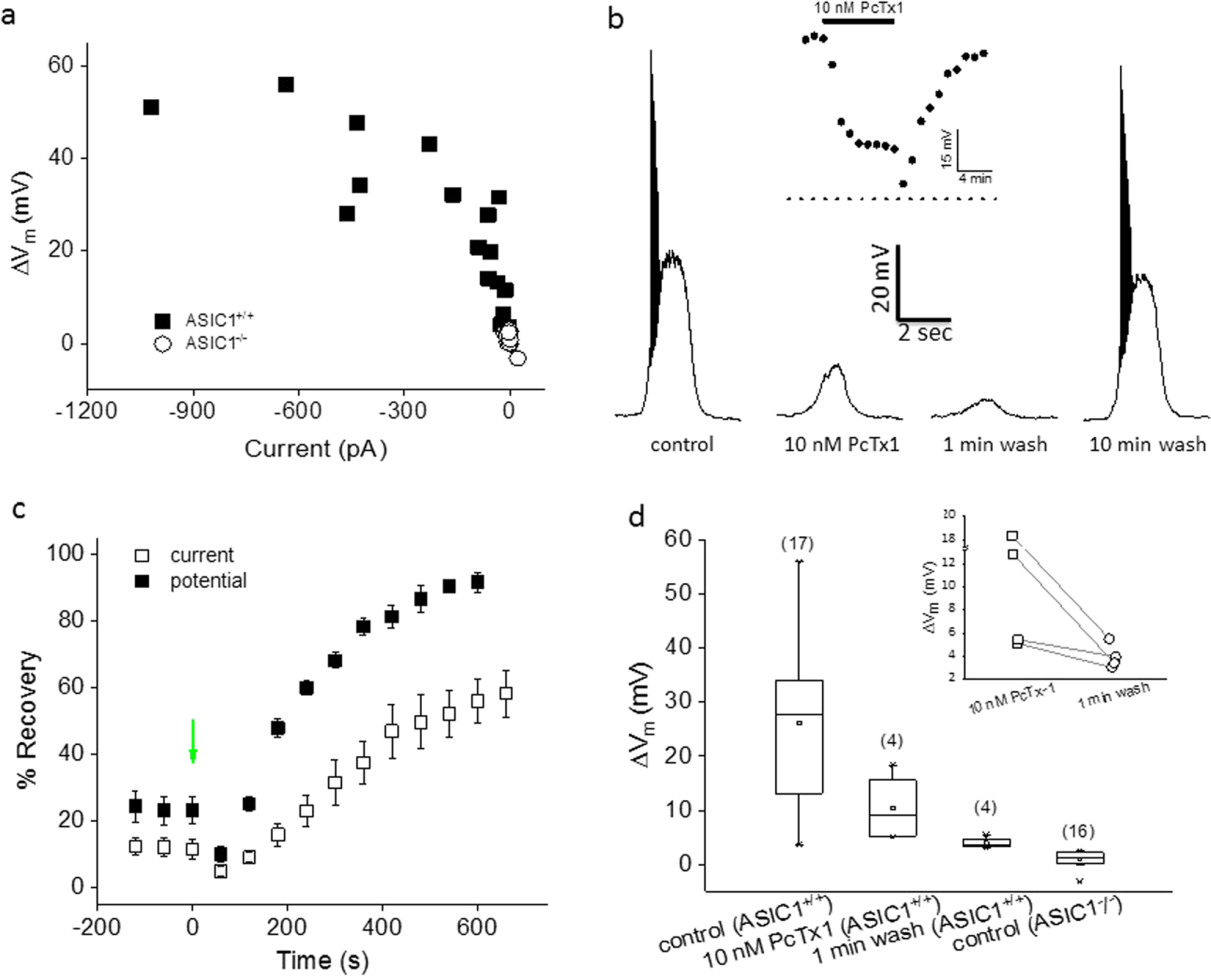
Potentiation of ASIC1a/2a by low concentration PcTx1 in mouse cortical neurons. (**a**) Correlation between pH6.8-induced current and membrane depolarization in wild-type (solid squares; n = 17) and ASIC1^−/−^ (open circles; n = 16) neurons. Each symbol represents one neuron. (**b**) Traces of pH6.8-induced membrane depolarization from a single neuron in the absence and presence of 10 nM PcTx1. Inset: Time course of the effect of 10 nM PcTx1 and wash. The dotted line at the bottom represents the baseline. (**c**) Time courses of pH6.8-induced responses upon removal of 10 nM PcTx1. Wash began at t = 0 sec as indicated by the green arrow. Open squares: current (n = 4); solid squares: membrane potential (n = 4). Responses were normalized to the control values for each cell just before application of 10 nM PcTx1. Note the initial lower responses (at t = 60 sec) than those in the presence of 10 nM PcTx1 (at t ≤ 0 sec). (**d**) Summary of pH6.8-induced membrane depolarization in wild-type and ASIC1^−/−^ neurons. Values for wild-type neurons during and upon initial wash of 10 nM PcTx1 were both significantly higher than that from ASIC1^−/−^ neurons (p < 0.05 and p < 0.0001, respectively). Inset: pair-wise comparison of pH6.8-induced membrane depolarization in the presence of 10 nM PcTx1 and 1 min after wash-off. The former was larger than the latter for all the four wild-type neurons tested, despite not reaching statistical significance (p = 0.11). A typical protocol used for experiments in Fig. 5 was as follows. For current recording, the holding potential was −80 mV; for membrane potential recording, a small hyperpolarizing current (0–100 pA) was injected to maintain the resting membrane potential near −70 mV. The conditioning pH was 7.4 and the test pH of 6.8 was applied for 2 sec before returning to pH7.4. This pH application protocol was repeated once every 60 sec. Where applicable, PcTx1 was present in both conditioning and test pH buffers for 4–8 min till reaching steady state before wash.

**Figure 6:**
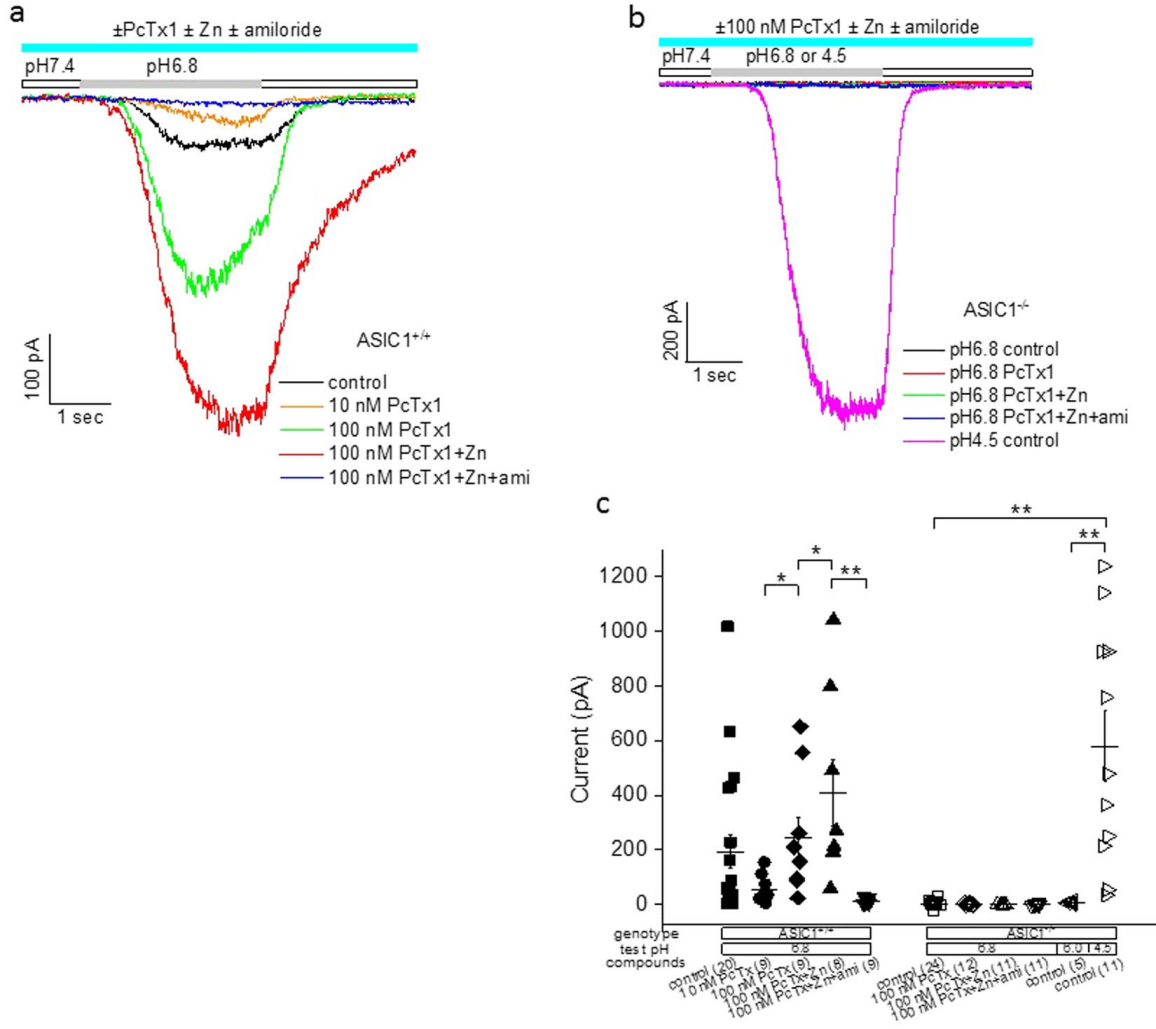
Potentiation of ASIC1a/2a by high concentration PcTx1 in mouse cortical neurons. (**a**) Traces of pH6.8-induced current from a single wild-type neuron in control buffer and in the presence of 10 nM PcTx1, 100 nM PcTx1, 100 nM PcTx1 + 300 μM Zn and 100 nM PcTx1 + 300 μM Zn + 500 μM amiloride. (**b**) Traces of pH6.8- or pH4.5-induced current from a single ASIC1^−/−^ neuron in control buffer (for both pH6.8 and pH4.5) and (for pH6.8 only) in the presence of 100 nM PcTx1, 100 nM PcTx1 + 300 μM Zn and 100 nM PcTx1 + 300 μM Zn + 500 μM amiloride. (**c**) Summary of pH6.8, pH6.0 or pH4.5-induced currents under conditions indicated for wild-type and ASIC1^−/−^ neurons. Numbers in the parentheses indicate the number of neurons tested under each indicated condition. The conditioning pH was 7.4 for all the experiments in Fig. 6.

We further examined the effects of PcTx1 on acid-induced responses in mouse cortical neurons. Wild-type (ASIC1^+/+^) neurons exhibited a good positive correlation between the amplitudes of pH6.8-induced currents and changes in membrane potential (Fig. 5a). In contrast, neither currents nor depolarization was evoked from ASIC1^−/−^ neurons in response to pH6.8 stimulation (Fig. 5a). These data indicated that virtually all the pH6.8-induced responses in wild-type neurons arose from ASIC1a-containing channels, given that ASIC1b is not expressed in cortical neurons. As with rat neurons, application of 10 nM PcTx1 to wild-type neurons resulted in substantial inhibition of pH6.8-induced depolarization, which was decreased further upon initial wash (Fig. 5b–d). Notably, the amplitudes of depolarization during and upon initial wash of 10 nM PcTx1 were both significantly higher than that from ASIC1^−/−^ neurons (p < 0.05 and p < 0.0001, respectively; Fig. 5d), suggesting that the depolarization in wild-type neurons in the presence of 10 nM PcTx1 involved ASIC1a. Since removal of PcTx1 should not result in decreased depolarization in wild-type neurons if ASIC1a were responsible for the depolarization in the presence of 10 nM PcTx1, the most logical candidate that fit this profile was ASIC1a/2a heteromeric channels. Perhaps the strongest argument in favor of this hypothesis came from the data shown in Fig. 6. In wild-type neurons, zinc (300 µM) increased 100 nM PcTx1-induced potentiation, which was completely blocked by amiloride (500 µM) (Fig. 6a and c); in contrast, neither PcTx1 nor PcTx1 + zinc evoked any current in response to pH6.8 stimulation in ASIC1^−/−^ neurons (Fig. 6b and c). There was also no pH6.0-induced responses in ASIC1^−/−^ neurons (Fig. 6c), further confirming the absence of functional ASIC1a/2a expression. On the other hand, pH4.5 evoked a current response in ASIC1^−/−^ neurons (Fig. 6b and c), indicating the presence of ASIC2a expression.

## Discussion

Of the ASIC subunits, ASIC1a and ASIC2a are the most abundantly expressed surface proteins in the CNS^23,24^. Expression of ASIC2b, though significant in the CNS^3^, appears restricted mainly to intracellular compartments (see ref.^27^; however, see also ref.^29^). Colocalization of ASIC1a and ASIC2a subunits results in significant expression of functional ASIC1a/2a heteromers in CNS neurons^20,23–26^.

Several venom-derived peptides have been shown to modulate ASIC channels, of which, PcTx1 is the most extensively studied^34,35^. The first-identified, most potent and significant effect of PcTx1 is the inhibition of ASIC1a homomeric channels^28^ by stabilizing the desensitized state^30^. PcTx1 has also been shown to potentiate ASIC1a activation at non-desensitizing pHs^30^. In addition, potentiation of homomeric ASIC1b^31,32^ and inhibition of heteromeric ASIC1a/2b^29^ have also been reported. PcTx1 was initially shown to be inactive at ASIC1a/2a^28^. However, those experiments were performed at pH7.4. A recent study^26^ reported significant inhibition when cells were held at pH6.95.

In this study, we reported novel pharmacology of PcTx1 at ASIC1a/2a, presenting strong evidence that PcTx1 had dual actions at the heteromeric channel, inhibition as well as potentiation. To our knowledge, this is the first report describing potentiating effects of PcTx1 on ASIC1a/2a, not only in a recombinant expression system, but also in neurons.

We first examined the exquisite pH dependence of PcTx1 inhibition of ASIC1a/2a. We showed that PcTx1 did not inhibit ASIC1a/2a at pH7.4, a non-desensitizing pH, but significantly inhibited the channel at a threshold pH (7.0) for desensitization, in agreement with the findings of Escoubas *et al*.^28^ and Joeres *et al*.^26^. We further extended these findings in two ways. First, we found that PcTx1 inhibited ASIC1a/2a with high affinity (IC_50_ = 2.9 nM at pH7.0), which is within an order of magnitude of its potency at ASIC1a. Second, this inhibition was incomplete at high concentrations of PcTx1, plateauing at ~75% in our recombinant expression system. The partial efficacy may be due to insufficient shifting by PcTx1 at pH7.0 of the ASIC1a/2a steady-state desensitization curve to reach full desensitization. Consistent with this idea, ASIC1a/2a was not completely desensitized in the absence of PcTx1 even at conditioning pH6.5 (Fig. 1c). An alternative scenario may also provide an explanation for the partial efficacy. PcTx1 binds at the interface between two ASIC1a subunits^36,37^, but has not been reported to interact with ASIC2a. It is possible that PcTx1 may bind with lower affinity to the 1a-2a-2a form of ASIC1a/2a which has no 1a-1a interface but two 1a-2a interfaces, leaving this fraction of the ASIC1a/2a channels substantially unbound/uninhibited in the range of PcTx1 concentrations we tested. In qualitative agreement with this scenario, Joeres *et al*.^26^ found that 50 nM PcTx1 inhibited 1a-2a-2a much less than 1a-2a-1a. Given the flexible stoichiometry of ASIC1a/2a assembly^6^, the ratio of the two forms of ASIC1a/2a heteromers (hence the PcTx1 efficacy) likely varies depending on the relative abundance of the ASIC1a and ASIC2a subunits. It is possible that both scenarios were responsible for the partial efficacy that we observed. Interestingly, PcTx1 is also a partial antagonist at ASIC1a/2b heteromers, at least under the conditions used in the study (notably, conditioning pH7.9)^29^.

Previously, PcTx1 has been shown to exhibit dual effects on ASIC1a homomeric channels. At conditioning pHs that are non-desensitizing, it potentiates channel activation. At near- or partially-desensitizing conditioning pHs, it promotes channel desensitization. In both cases, PcTx1 modulates ASIC1a by increasing the apparent affinity of the respective channel gating for protons^30^. We were intrigued by the analogy between ASIC1a and ASIC1a/2a in the conditions for PcTx1 inhibition to occur – channel desensitization. We reasoned that if the mechanism of PcTx1 inhibition was shared by the two channels, then it would seem plausible that it could be shared for PcTx1 potentiation. This would suggest that under conditions analogous to those for ASIC1a, PcTx1 should also potentiate ASIC1a/2a, which had not been reported. In this scenario, conditions favorable to observing potentiation of ASIC1a/2a by PcTx1 would seem to include at least the following: (1) at a non-desensitizing conditioning pH and (2) at a relatively moderate stimulating pH, such as a threshold, or even sub-threshold, activation pH.

Our initial attempts with experimental conditions (conditioning pH7.4 which was a non-desensitizing pH for ASIC1a/2a, test pH6.8 which was a sub-threshold pH for ASIC1a/2a activation, and 10 nM PcTx1; in CHO cells stably expressing ASIC1a/2a) generated sufficiently tantalizing results suggestive of the existence of such potentiation. One of the early clues came from the observation that pH6.8-induced depolarization in the presence of 10 nM PcTx1 was always decreased upon initial PcTx1 wash-off. This could not have been an effect on ASIC1a homomers co-expressed with ASIC1a/2a heteromers in the CHO cells, as PcTx1 should have inhibited ASIC1a under these conditions and removal of PcTx1 should not have resulted in additional inhibition at any time point. Furthermore, 10 nM PcTx1 should have completely inhibited ASIC1a in CHO cells (Fig. S2), rendering no additional inhibition possible. Indeed, no decrease in pH6.8-induced responses was observed upon PcTx1 removal in CHO cells stably expressing only ASIC1a homomers. Thus, the decrease in pH6.8-induced depolarization upon PcTx1 removal (1) was not an effect on ASIC1a homomers and (2) suggested an effect of potentiation in the presence of PcTx1. The fact that this wash effect occurred in all three ASIC1a/2a expression systems we recorded from (CHO cells exogenously expressing ASIC1a/2a, rat and mouse neurons) lends support to the hypothesis of ASIC1a/2a involvement. A second conclusion that could be drawn from these wash experiments was that this was a potent effect, with the threshold concentration of PcTx1 for potentiation being <10 nM. Indeed, this was directly observable in those CHO cells and neurons in which expression of homomeric ASIC1a was relatively low compared to that of ASIC1a/2a. In these cells (see examples in Supplementary Fig. S6), the pH6.8-induced current was increased upon switching from control buffer to that containing 10 nM PcTx1.

Potentiation of ASIC1a/2a was more pronounced at stimulating pHs near the activation threshold, such as pH6.8, than at more acidic pHs, indicating that, as with ASIC1a, PcTx1 promotes ASIC1a/2a channel activation by increasing the affinity of the channel for protons. Indeed, potentiation was all but non-existent as the pH was decreased to 6.0 or lower, a pH range commonly used in studying ASIC1a/2a. This may have contributed to the fact that potentiation of ASIC1a/2a by PcTx1 has not been reported in the literature.

As with ASIC1a/2a exogenously expressed in CHO cells, ASIC1a/2a in neurons was also potentiated by PcTx1. First, potentiation only occurred in wild-type neurons but was absent in ASIC1^−/−^ neurons, indicating that the ASIC1a subunit was involved (ASIC1b is only significantly expressed in peripheral neurons). Second, zinc further augmented the PcTx1 potentiation of the pH6.8-induced response, indicating an involvement of the ASIC2a subunit. Third, ASIC2a homomeric channels were not activated by pH6.8 with or without PcTx1 and ASIC2b is not activated by acidification^3^. Thus, neither ASIC2a nor ASIC2b homomers could be the target of the PcTx1 potentiation. Finally, ASIC1a/2b could not be responsible for the PcTx1 potentiation because (a) the effect of PcTx1 and zinc on ASIC1a/2b are both inhibitory^29^ and (b) at conditioning pH7.0, ASIC1a/2b is fully desensitized and unavailable for activation^29^, in contrast to our observation that transient application of PcTx1 still potentiated pH6.8-induced current (see Supplementary Fig. S3).

During normal neuronal activity in the brain, the amplitude and duration of acidification may be too small and/or transient for PcTx1 to produce a significant shift of the steady-state desensitization curve that results in inhibition of ASIC1a/2a. On the other hand, potentiation of ASIC1a/2a by PcTx1 is physiologically relevant in that it binds to the channel at the physiological pH and produces rapid potentiation of responses induced by very moderate pH drops. During synaptic transmission, for instance, protons released from presynaptic vesicles may cause a transient decrease in the synaptic pH sufficient to activate ASIC1a/2a^10^, at least in the presence of PcTx1. While ASIC1a/2a was not activated at pH6.8 in our recombinant expression system, potential endogenous modulators, such as zinc, may be present at sufficiently high concentrations in the synapse to shift its activation threshold near or above pH6.8^23,38–42^. As our results demonstrated (Fig. 6), not only did PcTx1 potentiate pH6.8-induced currents in neurons, the effects of PcTx1 and zinc were also additive, further increasing the likelihood of PcTx1 potentiation *in vivo*.

PcTx1 has been widely used *in vitro* and *in vivo* as a selective ASIC1a antagonist to probe the function of ASIC1a in various biological states. Our results suggest that PcTx1 may also be a valuable tool for understanding the functional role of ASIC1a/2a. With the increasing recognition that PcTx1 modulates multiple ASIC channels, however, the utility of PcTx1 as a selective tool for ASIC1a may be overstated. Not only can PcTx1 bind potently to multiple ASIC channels, its functional effects on the same channel can also vary greatly depending on the conditioning and stimulating pHs, as our current study demonstrated. Therefore, extra caution is warranted when designing and interpreting PcTx1 experiments. Our findings further expand the diverse and complex pharmacology of PcTx1 and contribute significantly to the understanding of the modulation of ASIC channels by PcTx1.

## Methods

### Stable cell lines and cell culture

Rat ASIC1a and ASIC2a channel cDNA constructs were assembled from published sequences, cloned into mammalian expression vectors (rat ACCN2 accession #NM_024154, in pCMV6-Entry; rat ACCN1 accession #NM_001034014, in pcDNA3.1; OriGene, Rockville, MD, USA), and individually transfected in Chinese Hamster Ovary (CHO) cells (Lipofectamine 2000, Invitrogen, Carlsbad, CA, USA). Clonal cells stably expressing ASIC1a or ASIC2a were selected based on resistance to G418 (ASIC1a) or zeocin (ASIC2a). The ASIC1a/2a stable cell line was obtained by transfecting the rat ASIC2a cDNA in CHO cells stably expressing rat ASIC1a and subsequently selecting for clones with resistance to both G418 and zeocin. Special attention was paid to ensure that the selected ASIC1a/2a clone had relatively low expression of ASIC1a homomers compared to that of ASIC1a/2a (based on pH6.8- and pH6.0-induced whole-cell currents, respectively). Cells were cultured in Ham’s F12, supplemented with 10% FBS, 1% penicillin-streptomycin and 500 µg/mL G418 (ASIC1a), 300 µg/mL zeocin (ASIC2a) or 600 µg/mL G418 and 300 µg/mL zeocin (ASIC1a/2a), and incubated at 37 °C with 5% CO_2_.

### Neuronal cultures

Rat cortical neurons were isolated from E-18 Sprague-Dawley rats (Charles River). Cortices were isolated and rapidly removed from decapitated pups and stored in ice-cold Hibernate-E media. Cortices were then transferred into a 15 mL conical tube and incubated at 37 °C for 10 min in 2 mL papain dilution buffer (5 mM L-cysteine, 5 mM EDTA, 10 mM HEPES and 100 µg/mL BSA) plus 1% papain (Worthington) and 0.1% DNase I (Thermo Fisher Scientific). Heat-inactivated FBS (2 mL) and additional 5 µL DNase I were added before cells were mechanically dissociated until homogeneous using a 10 mL pipette. After centrifugation (5 min at 1200 rpm at room temperature), the cell pellet was resuspended in culture media (Neurobasal medium supplemented with B27 and 0.5 mM Glutamax). Resuspended cells were centrifuged and the pellet was resuspended again in culture media. Cells were then filtered, centrifuged, resuspended, and plated at a density of 10^5^ cells per glass coverslip coated with poly-D-lysine in 24-well plates and maintained in culture media at 37 °C and 5% CO_2_. One half of the culture media was replaced with fresh culture media 2 days after plating. Mouse cortical neurons were isolated from E-17 or E-18 pups (Jackson Laboratory) and cultured using the same procedures/media as for rat neurons. ASIC1^−/−^ mice were obtained through a licensing agreement with Dr. John Wemmie’s lab (Univ. of Iowa). All procedures and experiments involving animals were carried out in accordance with internationally accepted guidelines for the care and use of laboratory animals in research. Animal protocols were approved by the Institutional Animal Care and Use Committee of Janssen Research & Development, L.L.C.

### Electrophysiology

CHO cells for use in manual patch-clamp electrophysiology were plated at low density onto glass coverslips and maintained in appropriate culture media for ASIC1a, ASIC1a/2a and ASIC2a, respectively. On the day of experiment, glass coverslips were placed in a chamber on the stage of an inverted microscope and perfused (∼1 mL/min) with an extracellular solution containing: 137 mM NaCl, 2 mM CaCl_2_, 5.4 mM KCl, 1 mM MgCl_2_, 5 mM glucose, 10 mM HEPES, pH 7.4, 310 mosM/L. Extracellular solutions at acidic pHs were made by titrating the pH7.4 solution with HCl and MES (for solutions at pH6.0 and lower). Pipette electrodes were filled with an intracellular solution of the following composition: 135 mM KCl, 4 mM MgATP, 0.3 mM Na_2_GTP, 10 mM EGTA and 20 mM HEPES, pH 7.2, 290 mosM/L. Neurons used for patch clamp recording were between 10 and 26 days in culture (DIV 10–26). The extracellular and intracellular solutions were the same as for CHO cells. In addition, the following cocktail was also added to the extracellular solution: 50 µM AP5, 20 µM NBQX and 20 µM bicuculline.

All recordings were conducted at room temperature (~22 °C) using an Axopatch 200B amplifier and pClamp 10 software (Molecular Devices). Currents or membrane potentials were measured using the whole-cell configuration of the patch clamp technique, digitized at 10 kHz and lowpass filtered at 2 kHz (currents) or 5 kHz (membrane potential). Series resistance was compensated at 75%. Responses were elicited by rapid perfusion of acidic solutions using the SF-77B Fast-Step Perfusion device (Warner Instruments, Hamden, CT, USA) for 300 ms (unless indicated otherwise) once every 30 s (CHO cells) or for 3 s once every 60 s (neurons) and allowed to stabilize and reach steady state in control and compound applications. For voltage-clamp experiments, the holding potential was −80 mV for neurons and 0 mV for CHO cells (unless indicated otherwise). PcTx1 was either included in or removed from both conditioning and test pHs concurrently, except for the experiments in Figs 3 and S3 in which PcTx1 was only included in the test pH buffer.

PcTx1 was purchased from Alomone Labs (Israel). All other chemicals were from Tocris (Minneapolis, MN, USA).

### Data Analysis

Baseline values (i.e., current or membrane potential amplitudes at the conditional pH) were subtracted to obtain responses evoked by the test pH. Responses were normalized for each cell before averaging (See figure legends for details of normalization for each type of experiment). Concentration–response data were fitted to a logistic function of the form: R = (A_1_-A_2_)/(1 + (C/C_0_)^h^) + A_2_, where R was the normalized response, C was either pH or PcTx1 concentration, C_0_ was the pH/concentration at which half-maximal response occurred (pH_50_, EC_50_ or IC_50_), h was the Hill coefficient, and A_1_ and A_2_ were constants. Data fitting was performed using Origin (Northampton, MA, USA). Data were reported as mean ± SEM, which resulted from independent measurements on n different cells. Statistical analyses were performed using the Student’s t-Test.

### Data Availability

The datasets generated during and/or analysed during the current study are available from the corresponding author on reasonable request.

## Electronic supplementary material

Supplementary Information
