# Supplementary material for: Dual actions of Psalmotoxin at ASIC1a and ASIC2a heteromeric channels (ASIC1a/2a)
Source: Sci Rep. 2018 May 8;8:7179. doi: 10.1038/s41598-018-25386-9 (PMC5940917; doi:10.1038/s41598-018-25386-9)
Supplement: Supplementary file 1 — Supplementary Information [file 41598_2018_25386_MOESM1_ESM.pdf]

Dual actions of Psalmotoxin at ASIC1a and ASIC2a heteromeric channels (ASIC1a/2a)

Yi Liu, Rebecca Hagan & Jeffrey Schoellerman

Neuroscience Discovery, Janssen Research & Development, L.L.C.,

3210 Merryfield Row, San Diego, CA 92121, USA

Supplementary Fig. S1

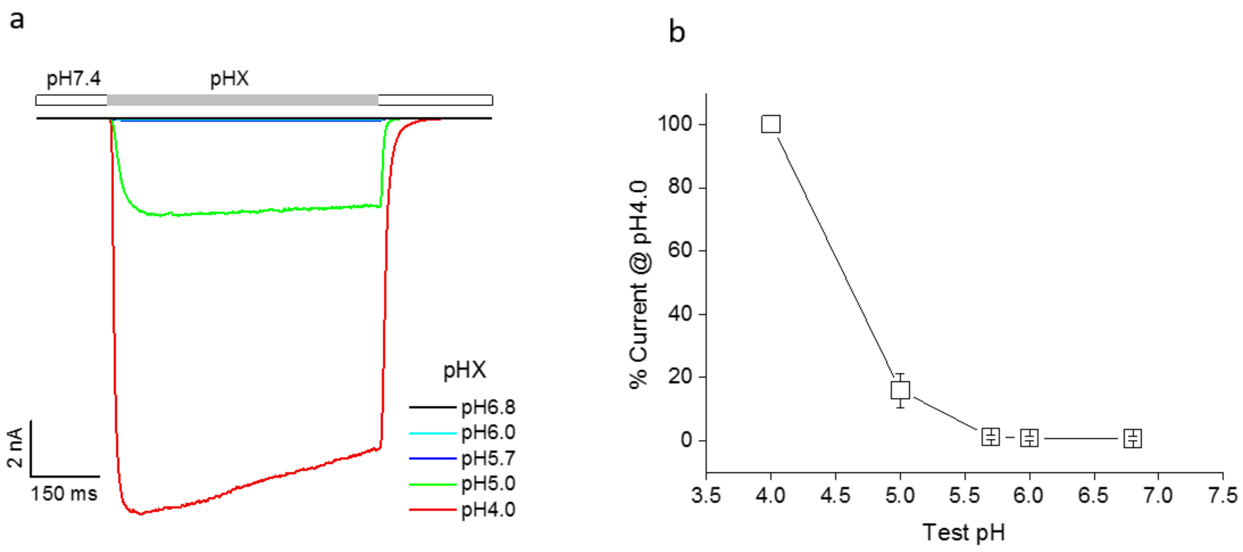

Supplementary Figure S1. pH dependence of activation of rASIC2a homomeric channels stably expressed in CHO cells. (a) Traces of H^+-induced currents from a single cell stably expressing only ASIC2a homomers. The solid horizontal bar indicates the period during which the test pH buffer (X=6.8, 6.0, 5.7, 5.0 or 4.0) was applied. (b) Average (n=3-6) pH dependence of ASIC2a activation. Responses were normalized to that at pH4.0 for each cell before averaging. The conditioning pH was 7.4 and holding potential was -80 mV.

Supplementary Fig. S2

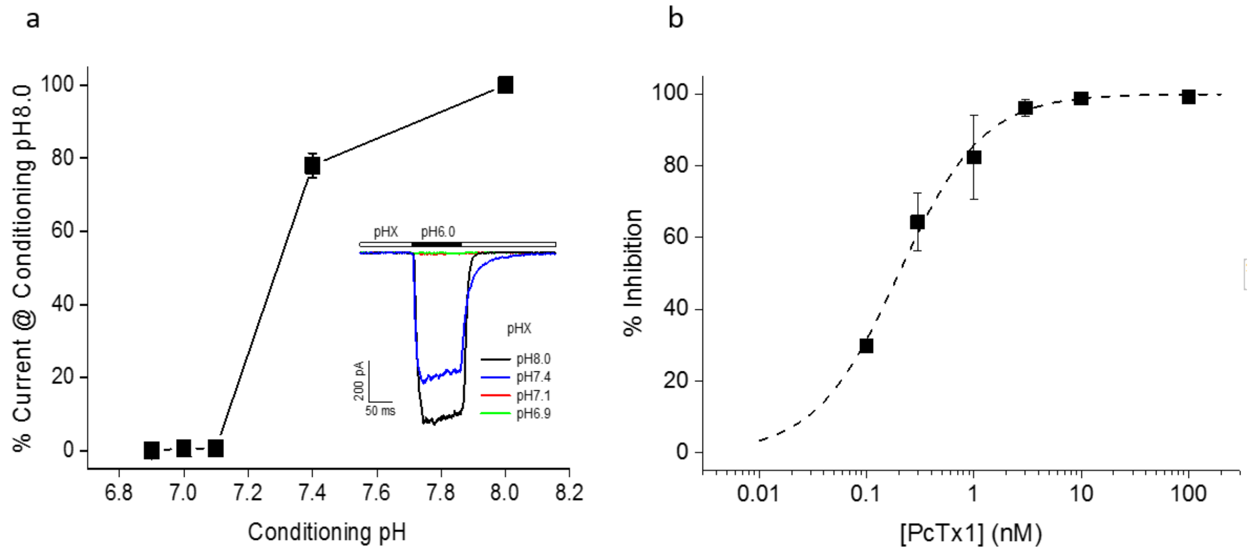

Supplementary Figure S2. Steady-state desensitization and PcTx1 inhibition of rASIC1a homomeric channels stably expressed in CHO cells. (a) Dependence of pH6.0-induced current responses on conditioning pH (n=4). Responses were normalized to that at conditioning pH8.0 for each cell before averaging. Inset: Current traces from a single cell showing pH6.0-induced current responses at various conditioning pHs (X=6.9, 7.1, 7.4 or 8.0) as indicated. (b) Concentration dependence of PcTx1 inhibition of ASIC1a (IC_{50}=0.20 µM; n=3). The conditioning pH was 7.4 and holding potential was -80 mV.

Supplementary Fig. S3

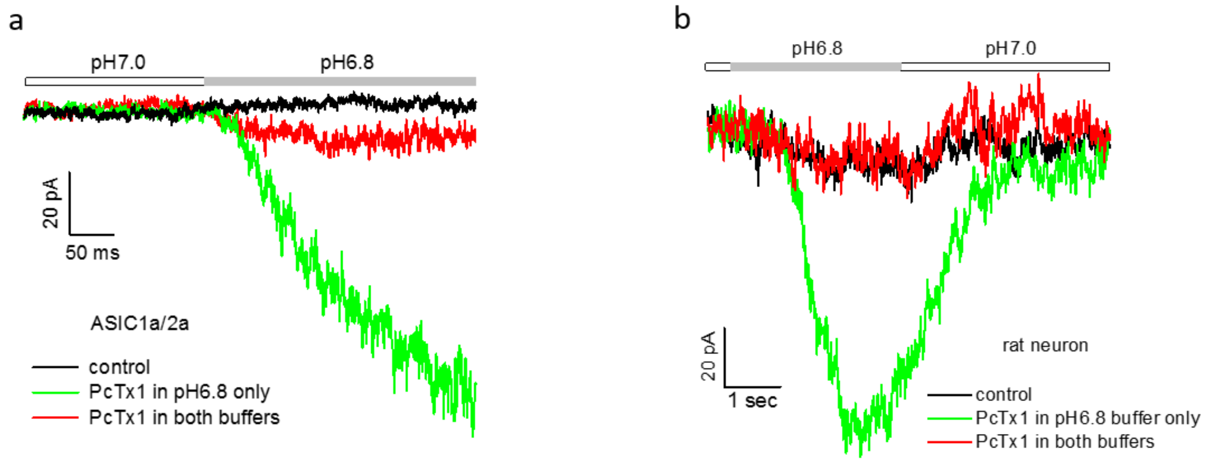

	pH6.8-induced current amplitude (pA)					
	rASIC1a/2a in CHO			rat cortical neuron		
	mean	se	n	mean	se	n
control	1.7	1.7	7	6.1	0.4	3
100 nM PcTx1 in pH6.8 buffer only	344.3	107.0	5	84.9	15.6	3
100 nM PcTx1 in both buffers	16.3	3.4	3	7.2	1.5	3

Supplementary Figure S3. Comparison of preincubation and transient application of PcTx1. (a) Traces of currents induced by the application of pH6.8 buffer from an ASIC1a/2a-expressing CHO cell. The holding potential was 0 mV. (b) Traces of pH6.8-induced current from a rat cortical neuron. The holding potential was -80 mV. Note the much larger pH6.8-induced current in both (a) and (b) with transient application of PcTx1 than that with preincubation. The holding pH was 7.0 for all the experiments in Fig. S3.

Supplementary Fig. S4

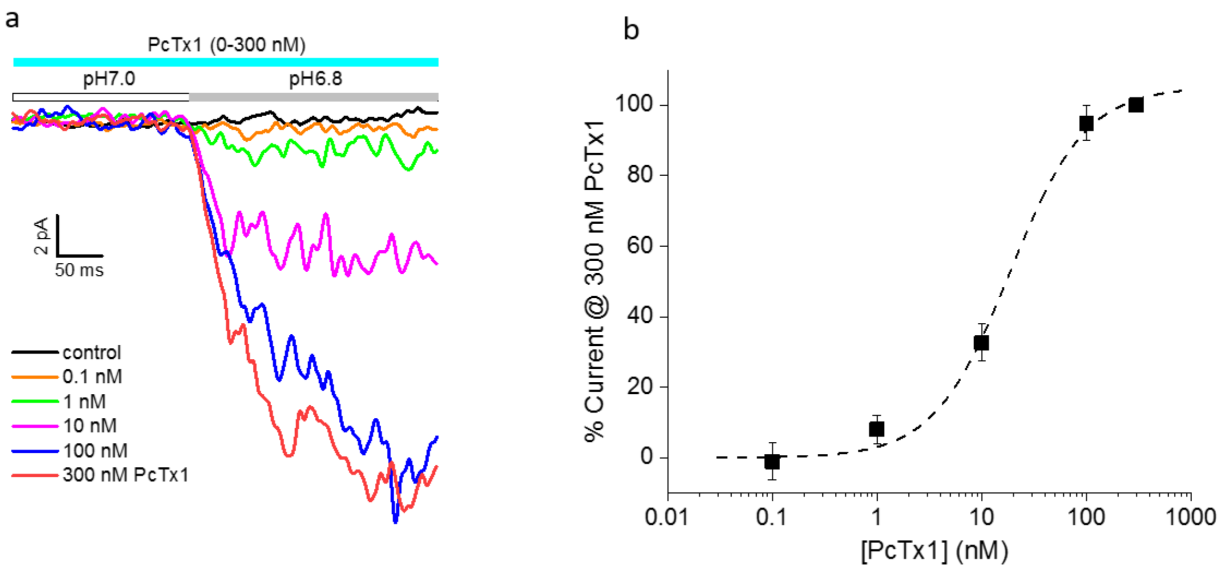

Supplementary Figure S4. Potentiation of ASIC1a/2a by PcTx1 under conditions of PcTx1 inhibition in CHO cells. Consistent with the partial efficacy of antagonism (Fig. 1f), potentiation of ASIC1a/2a by PcTx1 could still be observed with preincubation at the conditioning pH of 7.0, despite predominantly inhibitory actions of PcTx1 at this pH. (a) Traces of pH6.8-induced current from a single cell held at pH7.0 showing PcTx1 potentiation in the presence of 0.1-300 nM PcTx1. Note the expanded current scale as a result of the fact that only a small fraction of the pH6.8-induced current in the presence of PcTx1 was uninhibited and observable. (b) Average pH6.8-induced responses (n=4-6) as a function of PcTx1 concentration. Responses were normalized for each cell to the value at 300 nM PcTx1 before averaging. EC_{50} = 18.5 nM from the best fit (dashed line) to the data. The conditioning pH was 7.0.

Supplementary Fig. S5

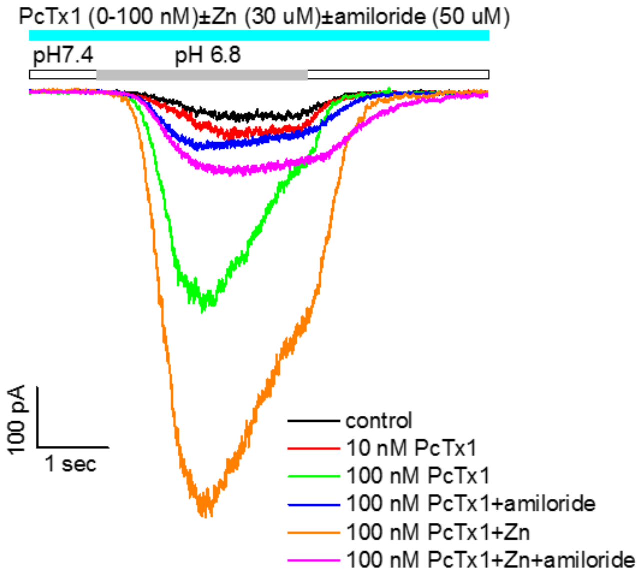

	current ratio (%)		
	mean	se	n
100 nM PcTx1/10 nM PcTx1	490.1	63.5	10
100 nM PcTx1+30 uM Zn/100 nM PcTx1	479.9	219.0	3
100 nM PcTx1+30 uM Zn+50 uM amiloride/100 nM PcTx1+30 uM Zn	20.8	3.1	3

Supplementary Figure S5. Effects of Zn and amiloride on PcTx1 potentiation of pH6.8-induced current in rat cortical neurons. Top: Traces of pH6.8-induced current from a single neuron showing (1) current potentiation by PcTx1 (100 nM), (2) augmentation of the PcTx1-potentiated current by Zn (30 uM), and (3) inhibition of the currents in (1) and (2) by amiloride (50 uM). Bottom: Table summarizing the results from multiple neurons. The conditioning pH was 7.4.

Supplementary Fig. S6

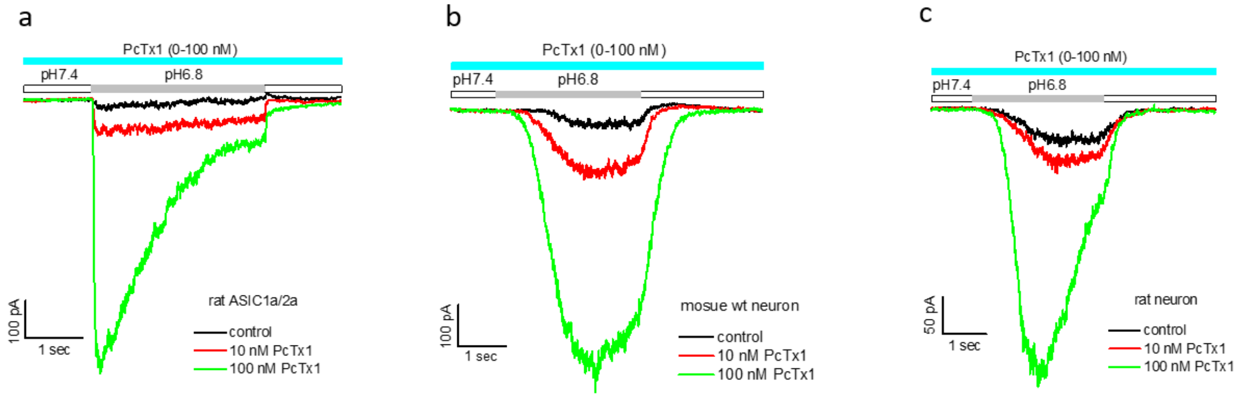

Supplementary Figure S6. Examples of cells in which pH6.8-induced current in the presence of 10 nM PcTx1 was larger than that in control buffer. (a) A CHO cell stably expressing ASIC1a/2a. (b) A wild-type mouse cortical neuron. (c) A rat cortical neuron. The conditioning pH was 7.4 for all the experiments.

Supplementary Table 1

		% Current @ pH5.7 Control		
Test pH	[PcTx1] (nM)	mean	se	n
6.8	0	0.1	0.0	5
6.8	100	8.1	4.3	3
5.7	100	107.7	0.5	3

Supplementary Table 1. Preferential potentiation of ASIC1a/2a by PcTx1 at moderate stimulating pHs in CHO cells. The conditioning pH was 7.4. PcTx1 was present in both the conditioning and test pH buffers. Acid-induced peak currents were normalized for each cell to that induced by pH5.7 control buffer in the absence of PcTx1. Cells selected for these experiments had negligible homomeric ASIC1a expression.
